# Glycosylation and Lipidation Strategies: Approaches for Improving Antimicrobial Peptide Efficacy

**DOI:** 10.3390/ph16030439

**Published:** 2023-03-14

**Authors:** Rosa Bellavita, Simone Braccia, Stefania Galdiero, Annarita Falanga

**Affiliations:** 1 Department of Pharmacy, School of Medicine, University of Naples ‘Federico II’, Via Domenico Montesano 49, 80131 Naples, Italy; 2 Department of Agricultural Sciences, University of Naples ‘Federico II’, Via dell’ Università 100, 80055 Portici, Italy

**Keywords:** antimicrobial peptides, chemical strategies, glycosylation, lipidation

## Abstract

Antimicrobial peptides (AMPs) have recently gained attention as a viable solution for combatting antibiotic resistance due to their numerous advantages, including their broad-spectrum activity, low propensity for inducing resistance, and low cytotoxicity. Unfortunately, their clinical application is limited due to their short half-life and susceptibility to proteolytic cleavage by serum proteases. Indeed, several chemical strategies, such as peptide cyclization, *N-*methylation, PEGylation, glycosylation, and lipidation, are widely used for overcoming these issues. This review describes how lipidation and glycosylation are commonly used to increase AMPs’ efficacy and engineer novel AMP-based delivery systems. The glycosylation of AMPs, which involves the conjugation of sugar moieties such as glucose and *N-*acetyl galactosamine, modulates their pharmacokinetic and pharmacodynamic properties, improves their antimicrobial activity, and reduces their interaction with mammalian cells, thereby increasing selectivity toward bacterial membranes. In the same way, lipidation of AMPs, which involves the covalent addition of fatty acids, has a significant impact on their therapeutic index by influencing their physicochemical properties and interaction with bacterial and mammalian membranes. This review highlights the possibility of using glycosylation and lipidation strategies to increase the efficacy and activity of conventional AMPs.

## 1. Introduction

The last decade has seen an increase in the improper use of antibiotics, resulting in a rise in antibiotic-resistant infections [1,2]. Pathogens belonging to the ESKAPE group, including *Enterococcus faecium, Staphylococcus aureus, Klebsiella pneumoniae, Acinetobacter baumannii, and Pseudomonas aeruginosa*, are responsible for causing multidrug-resistant infections. The difficulty in treating these infections has led to high levels of morbidity and mortality, making them a global concern [3,4].

**Figure 1:**
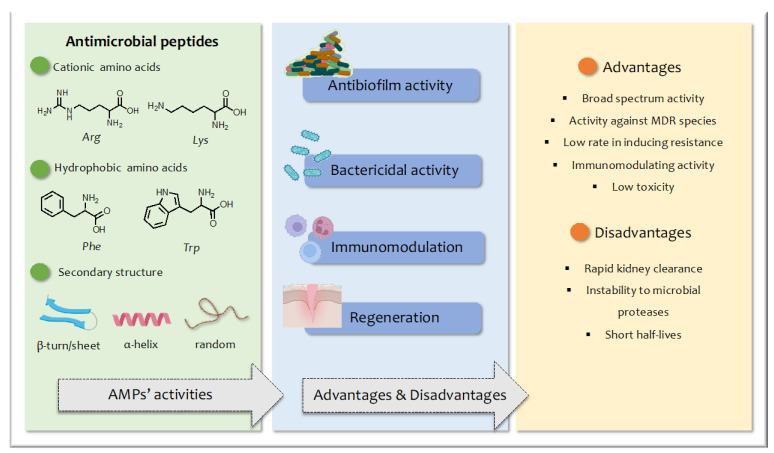
Schematic representation of advantages and disadvantages of AMPs and their structural features and biological activity.

Antimicrobial peptides (AMPs) have emerged as new drug candidates and have attracted the attention of researchers as the most viable replacement for conventional antibiotics [5]. AMPs are host defense molecules that play a crucial role in the innate immune response and exhibit significant efficacy against a broad spectrum of pathogens, including Gram-positive and Gram-negative bacteria and fungi [6,7,8,9,10]. AMPs are generally composed of up to 40 amino acid residues, are characterized by a positive net charge due to the presence of cationic residues (e.g., Arg, Lys), and are amphipathic thanks to the simultaneous presence of hydrophobic residues (e.g., Phe, Trp) (Figure 1). They are typically classified based on their secondary structures such as β-sheet, α-helical, loop, and extended peptides [11]. The ratio between the hydrophilic and hydrophobic residues determines their amphiphilic features, which is crucial for their biological activity [12].

The mode of action of AMPs varies widely and includes direct permeabilization and disruption of the bacterial membrane, binding with intracellular targets, and immunomodulation of the host [13]. This mechanism of action differs from most conventional antibiotics, which generally act on specific cellular targets [14], making it more difficult for bacteria to develop resistance [15]. Furthermore, evidence has shown that AMPs can cross the bacterial membrane and reach intracellular targets such as DNA and RNA or induce the production of reactive oxygen species that cause cell damage. Moreover, AMPs can modulate the immune response or exhibit anticancer activity [16,17,18]. This diversity in their mode of action has generated great interest in the clinical potential of AMP-based therapies.

The main mechanism of action of AMPs is based on their interaction with the membrane of the pathogen [19]. Although the exact process is not yet fully defined, studies performed on model membranes (e.g., liposomes) have shown that the positive charge is responsible for the initial interaction with the negatively charged membrane of microorganisms, whereas the hydrophobic portion is responsible for their insertion into the lipid bilayer [20]. Consequently, achieving the right balance between the hydrophilic and hydrophobic domains is key in determining the specificity of action of AMPs [21]. The positioning on the surface of the membrane and/or the incorporation into the membrane causes the disruption of membrane integrity with pore formation or membrane permeabilization followed by osmotic shock and loss of homeostasis [22]. The hypothetical mechanisms of membrane action are generally classified into three models: (i) the barrel-stave model, where peptides, typically in their α-helical conformation, embed into the hydrophobic core of the bacterial membrane and interact with the polar head groups of phospholipids, inducing the formation of transmembrane pores and leading to bacterial cell death [23]; (ii) the carpet model, where AMPs position themselves on the membrane surface by inducing membrane disruption in a detergent-like manner [24]; and (iii) the toroidal-pore model, a mechanism similar to the barrel-stave model, where the peptide molecules fold inwards, leading to the formation of a toroidal-shaped pore [25]. In both the barrel-stave and the toroidal-pore models, transmembrane pores form spontaneously because of peptide aggregation on the membrane surface [26].

Another factor that plays a crucial role in the mechanism of action is undoubtedly the secondary structure assumed by AMPs. Usually, AMPs are characterized by conformational flexibility, changing their structure when moving from the aqueous solution to the lipid environment. Their structural flexibility is the driving force for the interaction. For instance, as observed in some AMPs isolated from the skin of Australian tree frogs, adsorption inside bacterial membranes is driven by electrostatic and hydrophobic interactions that cause conformational changes in the peptide [27].

As mentioned above, the interest in AMPs as alternative or combination therapies compared to traditional antibiotics stems from several key advantages, including their broad-spectrum activity, near absence of resistance issues, and potential synergistic effects when combined with other antibiotics [28]. Despite their potential, AMPs also present several limitations due to a number of issues. Firstly, natural AMPs are composed of L-amino acids easily recognized by proteases, causing rapid degradation and kidney clearance. Other disadvantages include high production costs and poorly understood pharmacokinetics and toxicity [29].

Indeed, many approaches have been investigated to circumvent these limitations given the possible therapeutic uses of AMPs. An understanding of the structure–function relationship has allowed the design of analogs of natural AMPs through chemical modifications and/or the use of delivery tools. Therefore, this review will focus on chemical modifications with an emphasis on the covalent conjugation of carbohydrates and lipid tails to optimize and enhance the potency and stability of AMPs, as well as to improve their delivery.

## 2. Structural Modification of AMPs

**Figure 2:**
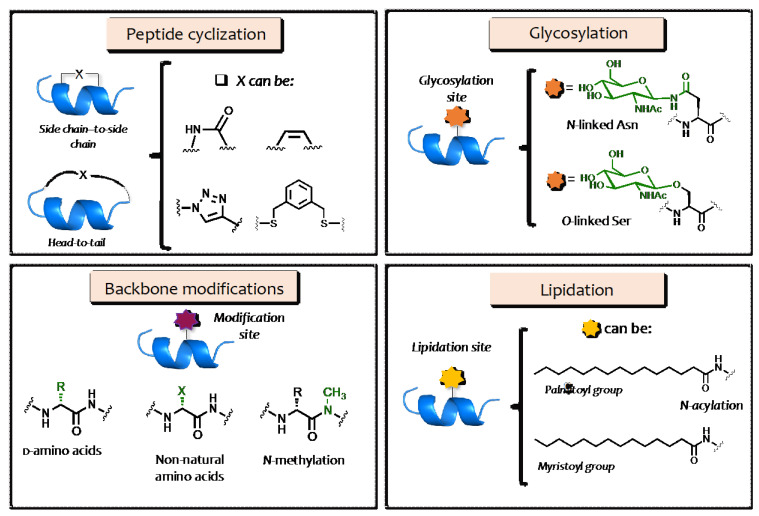
Schematic representation of the most common strategies used to improve the efficacy of AMPs, including cyclization, backbone modifications, glycosylation, and lipidation.

Approaches used to increase the activity, specificity, biocompatibility, and half-life of AMPs include the replacement of natural l-amino acids with their d-enantiomers or non-natural amino acids [30,31]; peptide cyclization, including side chain-to-side chain and head-to-tail peptide cyclizations [32,33,34,35]; and the *N-*methylation of the peptide backbone (Figure 2) [36]. For instance, the cyclization of linear peptides has been shown to reduce cytotoxicity toward mammalian cells while showing greater selectivity toward bacterial cells, demonstrating that linearity is not a prerequisite for the lytic activity of AMPs [37,38].

Other strategies include (i) PEGylation [39,40], which allows for greater solubility and stability in aqueous solutions while reducing renal clearance [41]; (ii) glycosylation involving carbohydrate conjugation, which broadens peptide diversity by extending their properties and functions [42]; and (iii) lipidation involving the covalent attachment of fatty acids, which affects peptide activity through several mechanisms [43,44].

Here, we describe glycosylation and lipidation as the most widely used strategies to enhance the efficacy, broad-spectrum activity, biocompatibility, and protease resistance of AMPs.

## 3. AMP Glycosylation

The glycosylation strategy consists of the covalent conjugation of a sugar moiety to the AMP sequence, likely promoting changes in their conformation and chemical, physical, and biological properties. In general, glycosylation increases the diversity of peptides and broadens their range of functionality. In fact, the incorporation of a glycan moiety has been shown to improve their antimicrobial potency and immunomodulation while also influencing their pharmacokinetic and pharmacodynamic properties.

The presence of the glycan unit increases the hydrophilicity and bioavailability of peptides, which improves their active transport through cell membranes by targeting glucose transporters located on the surface [45], induces specific conformations influencing their activity, and increases their resistance to enzymatic degradation, thus raising their half-life.

However, glycosylation does not always improve the antimicrobial potency of AMPs because the glycan conjugation may reduce both the hydrophobicity and overall positive charge of the peptides, which highly influences their interaction and insertion into the anionic membranes of bacteria [46].

**Figure 3:**
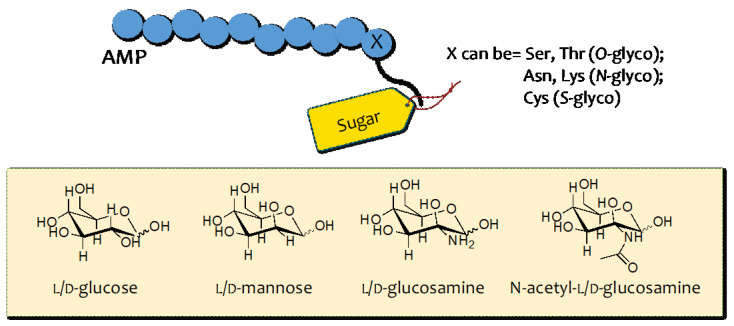
Examples of sugar moieties linked to AMP through the glycosylation strategy.

From a chemical point of view, the development of glycosylated peptides involves covalently linking glycans to a specific side chain of the amino acid residues present in the peptide sequence. Depending on the functional group implicated, glycosylation can be classified into four groups: *N-*glycosylation, *O-*glycosylation, *C-*glycosylation, and *S-*glycosylation [47]. In addition, the glycan composition, structure, and length determine the type of glycosylation. A variety of sugar moieties, including l/d-glucose, l/d-mannose, l/d-glucosamine, and *N-*acetyl- l/d-glucosamine (Figure 3), can be used and linked based on the designed AMP to improve its functionalities. As early as 20 years ago, several *O*-linked glycosylated and proline-rich AMPs derived from insects were identified such as diptericin, drosocin, formaecin, lebocin, and phyrrorricin [48]. Therefore, glycosylated AMPs represent only a small group of AMPs with broad-spectrum activity.

Glycosylation enhances the antimicrobial properties of AMPs, as well as their stability and biological properties both in vitro and in vivo. It also influences their physicochemical properties such as peptide rigidity, solubility, aggregation ability, and secondary structure [49,50,51,52]. Peptide stability is particularly important for the development of therapeutic peptides and glycosylation offers many opportunities for modifying the degree of glycosylation, glycan type, structural composition, and size. For instance, enfuvirtide is an antiretroviral drug that has been glycosylated with sialic acid residues to improve its half-life without affecting its sensitivity and activity toward the target [53].

### 3.1. O–Glycosylation

The O-glycosylation strategy involves covalently linking the glycosidic moiety to the serine (Ser) or threonine (Thr) side chains.

**Figure 4:**
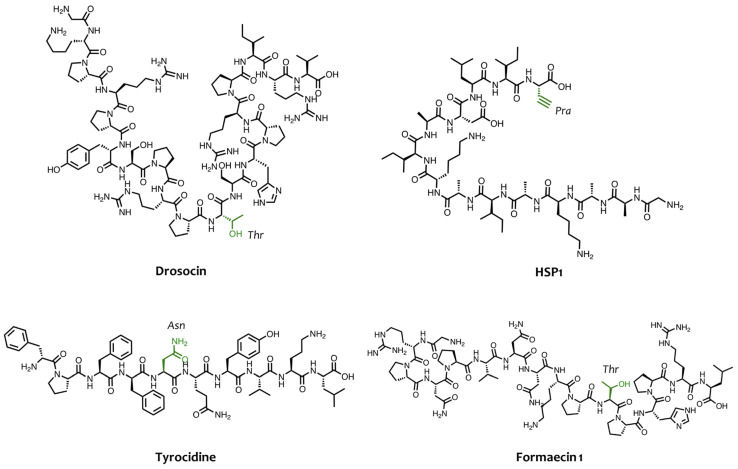
Chemical structures of some AMPs and residues implicated in the glycosylation reaction.

Depending on the kind of glycan moiety, this strategy can be further divided into *O-*mucin-type *O-*glycosylation, *O-*fucosylation, and *O-*glucosylation. Several studies have shown that *O-*mucin-type *O-*glycosylation, which involves the covalent attachment of *N-*acetylgalactosamine (GalNAc) residue to the hydroxyl (-OH) group of Ser and Thr, can enhance the antimicrobial activity of AMPs [54]. The importance of glycosylation in AMPs such as diptericin, formaecin, and the bacteriocin-family member, enterocin F4-9, is clearly supported by the fact that the absence of glycosylation eliminates their antimicrobial activity [55,56]. Some proline-rich AMPs are already *O-*glycosylated when isolated from natural sources and they are not active when synthesized without the GalNAc residue. For instance, Bulet and co-workers have isolated the glycosylated peptide drosocin (GKPRPYSPRPTSHPRPIRV) from *Drosophila* [57], which presents GalNAc and galactose residues on the Thr residue in position 11 (Figure 4), which are crucial elements for its antibacterial activity against *E. coli* D22 with a minimum inhibitory concentration (MIC) of 75 nM. In fact, synthetic derivatives lacking the *O-*glycosidic moieties exhibited a drastic loss of activity that was about 5–10 times lower against *E. coli* D22 and other Gram-negative strains [58,59]. However, the Thr residue of drosocin has undergone glycosylation screening by designing and synthesizing several analogs bearing different carbohydrate moieties [56]. In that study, Talat et al. performed *O-*glucosylation*, O-*mannosylation, and *O-*mucin-type *O-*glycosylation on the Thr residue, showing that the significant impact of glycosylation on activity and peptide conformation depended on the stereochemistry of the sugar. The authors also showed that β-linked sugars induced flexibility and unstrained conformations, unlike α-linked analogs that led to limited conformations with high stability [60]. These conformational changes had a drastic influence on the activity, with reduced activity observed when the anomeric sugar configuration changed from α- to β, confirming the dependence of glycosylation on the configuration of the sugar linkage [61].

Other examples of natural *O-*glycosylated AMPs are the peptides formaecin 1 (GRPNPVNNKPTPHPRL, Figure 4) and formaecin 2 (GRPNPVNTKPTPYPRL), with GalNAc residue *O-*linked, which were identified in the bulldog ant *Myrmecia gulosa*. Mackintosh and co-workers showed that the concentration of both non-glycosylated formaecins for killing *E. coli* was 75 times higher than their glycosylated isoforms, confirming the importance of GalNAc residue for their activity [62].

By applying the O-fucosylation strategy, Wu et al. developed a synthetic glycosylated peptide, namely HYL-33, which was derived from the native peptide HYL isolated from the venom of the Hylaeus signatus bee [63]. HYL-33 is a stapled peptide that is characterized by an L-fucose residue on Ser^4^ and showed strong activity against a variety of bacteria including S. lentus, E. faecalis, and A. johnsonii [64].

O-glycosylation is considered crucial for some insect AMPs, as in the case of the members of the bacteriocin family. For example, deglycosylation of the Ser and Thr residues of enterocin F4-9 caused a loss of activity toward *E. faecalis* and *E. coli* JM109 [65], showing that the GalNAc residue interacts with the target molecules in these susceptible bacteria.

Moreover, *O-*glycosylation has been proven efficacious in improving the activity of antifungal peptides. For example, Hassallidins, produced by cyanobacteria and other prokaryotes, are cyclic glycolipopeptides consisting of an eight-amino-acid residue peptide ring, where a fatty acid chain and sugar moieties are attached [66]. Hassallidins are highly selective against fungi because they showed antifungal activity against several opportunistic human pathogenic fungi but did not have any antibacterial activity. For example, the IC_50_ of Hassallidin D is 0.29–1.0 μM against *Candida* strains and acts by disrupting sterol-containing cell surface membranes [67].

Interestingly, O-linked glycosylation has also been shown to improve the blood–brain barrier (BBB) penetration of peptides such as opioid peptides [68], representing a promising strategy for targeting infections and facilitating the elimination of persistent infections from the nervous system.

### 3.2. N–Glycosilation

The asparagine (Asn) side chain represents a relevant *N-*glycosylation site to covalently attach carbohydrates and enhance AMP activity [69,70]. Monosaccharides, disaccharides, or lactose can be directly linked to Asn through solid-phase peptide synthesis using the Fmoc/tBu strategy [71]. Hu and co-workers designed and synthesized a series of cyclic *N-*glycosylated tyrocidine (Figure 4) derivatives bearing the glycans attached to the peptide backbone through different linkers. They showed how this strategy had a strong impact on the antibacterial activity of tyrocidine by increasing its efficacy against *Bacillus subtilis*, methicillin-resistant *S. aureus*, and vancomycin-resistant *Enterococcus* [72].

Plants are also a rich source of glycosylated AMPs, for instance, datucin contains a terminal GlcNac-asparagine and displays activity against both planktonic and biofilm *Candida albicans*, and even against multidrug-resistant clinical strains [73].

In addition, the lysine (Lys) amino acid is considered an N-glycosylation site as shown by Grimsey et al. [74]. In their study, the authors conjugated either glucose, N-acetyl glucosamine, galactose, mannose, or lactose residues to the synthetic AMP, peptide A1. In this case, the impact of N-glycosylation on the antimicrobial activity of peptide A1 against different microbial species was found to be negligible because the enhancement of hydrophilicity can lead to both a reduction in hydrophobicity and weaker interaction with lipopolysaccharides and teichoic acids of Gram-negative and Gram-positive bacteria, respectively.

### 3.3. S–Glycosilation

The free thiol group of the cysteine (Cys) side chain is the main site implicated in *S-*glycosylation [75], which has been shown to improve the peptide half-life in serum. Different studies performed on *S-*glycosylation showed an increase in the antimicrobial potency of several AMPs and antibiotics. For example, Amso et al. showed that the *S-*glycosidic linkage of the bacteriocin glycocin F offers a promising route for achieving more bioactive bacteriocins [76]. By applying the chemical ligation strategy and incorporating two GalNAc moieties on Cys in positions 18 and 43, the authors developed a more active glyco-mutant analog with enhanced activity (IC_50_ of 0.60 ± 0.10 nM) against *Lactobacillus plantarum*. However, a study conducted by Oman and co-workers identified the *S*-glycosylated sublacin peptide containing a glucose moiety attached to Cys^22^ [77], which is essential for its activity against a spectrum of Gram-positive species, including *B. subtilis*, *B. megaterium*, and *S. aureus*. Subsequently, Hsieh et al. synthesized two *S-*glycosylated sublacin analogs by replacing glucose with d-galactose and d-GalNAc moieties on Cys^22^ to understand the impact of the glycan on the secondary structure [78]. Both sugar moieties induced an α-helix structure, as observed by circular dichroism (CD) and nuclear magnetic resonance (NMR) spectroscopies.

### 3.4. C–Glycosylation

C-glycosylation is not the most widely used strategy for obtaining glycosylated AMPs, with just a few examples reported in the literature. C-glycosyl amino acids and peptides can be synthesized by employing copper(I)-catalyzed azide–alkyne 1,3-dipolar cycloaddition (CuAAc) [79,80] or via a nickel-catalyzed reductive hydroglycosylation reaction between alkyne and glycosyl bromide residues [81].

For example, Junior and co-workers synthesized two C-glycosylated derivatives of the antifungal hylaseptin-P1 peptide (HSP1, Figure 4) by performing the CuAAc reaction between the azide group linked to carbohydrate (GlcNac and Glc) and the alkyne present on backbone peptide [82]. Biologically, these glycosylated peptides not only displayed better antifungal activity against *Candida albicans* but also promoted a strong inhibition of ergosterol biosynthesis due to the presence of triazole and sugar moieties in comparison to the deglycosylated peptide HSP1.

In addition, the C-glycosylation strategy has demonstrated promise and efficacy in enhancing the anti-HIV activity of the peptide C34 (IC_50_ of 21 nM) derived from the C-terminal ectodomain of gp41 [83]. In this case, the presence of triazole and monosaccharide GlcNac was found to improve both the activity and resistance against protease and glyco-amidase-catalyzed digestion.

## 4. AMP Lipidation

**Figure 5:**
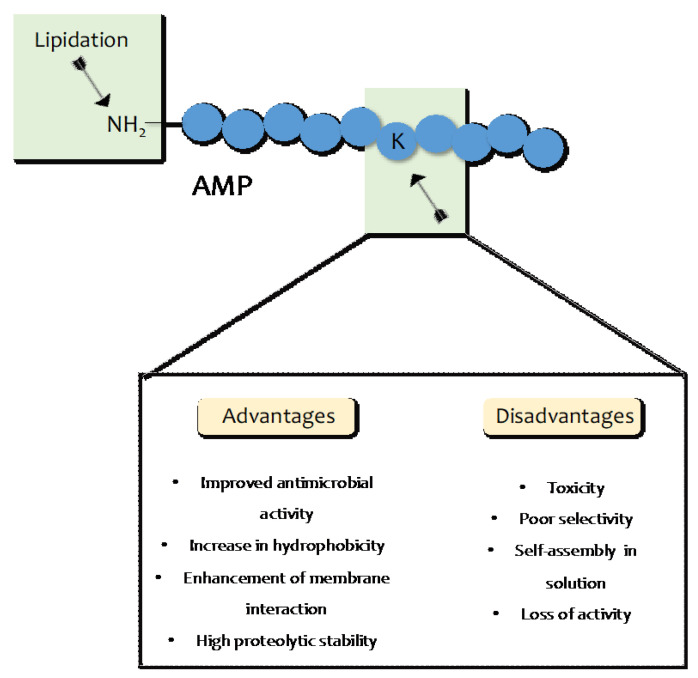
Advantages and disadvantages of lipidation strategy.

Another strategy to improve the antimicrobial potency of AMPs without causing structural modifications to their properties is lipidation, which involves the attachment of a fatty acid moiety to *N-*terminal residues or lysine side chains [84,85]. The incorporation of lipid tails of different lengths enhances the hydrophobicity of AMPs and confers better membrane interaction, better permeability, and protection against enzymatic proteolysis (Figure 5) [86,87,88]. It is likely that the improved potency is correlated to the length of the acyl chain, which also influences their pathogenic specificity [88] and enhances the interactions between the bacterial cell membrane and the fatty acid conjugated on the peptide. As acyl chains grow in length, there is an increased tendency for self-assembly in an aqueous solution, which determines a loss of peptide interaction with bacterial or fungal membranes.

As demonstrated by Húmpola et al., lipopeptides with longer chains of C17 and C20 were not active against bacteria and *Candida* species, probably due to their strong ability to self-assemble, whereas the best activity was observed for lipopeptides with fatty acids of C8 and C14 with an MIC of between 0.7 and 5.8 μM [89].

When the fatty acids are conjugated to AMPs, it is also crucial to preserve their net positive charge to ensure their binding and bacterial interaction. Indeed, the conjugation of the lipid tails of C13, C10, C7, and C5 at the *N-*terminus of the peptide [Pro^3^,DLeu^9^,DLys^10^]TL derived from the natural Temporin L peptide caused a reduction in the net positive charge from +4 to +3, with a consequent reduction in the activity of lipopeptides toward *S. aureus*, *K. pneumoniae,* and *P. aeruginosa.* In [90,91], the authors identified the para position of Phe^1^ as the optimal position for the attachment of fatty acids, as it preserved the positive charge +4 and demonstrated the best activity against both bacteria and *Candida* species.

In addition, the length of the conjugated fatty acids may increase hydrophobicity, enhancing the selectivity toward mammalian cells with consequent toxicity. Thus, although the increase in the hydrophobicity of peptides can improve antimicrobial activity, it is crucial to preserve the right hydrophilicity/hydrophobicity balance to avoid an increase in toxicity. The general toxicity is its direct dependence on the acyl chain length because longer acyl chains cause greater hemolysis. As mentioned previously, this trend is likely due to the high affinity and insertion of the long acyl chains of lipopeptides into the eukaryotic membrane. Clearly, a well-chosen chain length is key to determining the balance between improved antibacterial properties and selectivity.

The optimal chain lengths are most commonly C8–C12, which seem to be the most beneficial to secondary structure formation and membrane insertion. Indeed, the conjugation of fatty acids to the synthetic peptide CG117-136 induced higher α-helical content compared to the native peptide in the presence of a membrane-mimetic environment, favoring an enhanced insertion into liposomes and thus a higher ability to disrupt the bacterial membrane [92].

In addition, several studies involved ultrashort lipopeptides with potent antimicrobial activity against a variety of microorganisms. Makovitzki and co-workers synthetized peptides composed of only four D,L amino acids conjugated to aliphatic acids with different chain lengths [93]. Lohan et al. reported a library of small cationic ornithine-based non-natural lipopeptides by varying the content of both the cationic charge and hydrophobic bulk and analyzed their antimicrobial activity [94,95]. Interestingly, despite their short peptide chain length, their mode of action was similar to that of AMPs, providing great economic advantages.

Another interesting study performed by Sikora et al. showed the significant impact of glycosylation on a library of ultrashort lipopeptides. In particular, three different fatty acids (C14, C16, C18) were attached to the *N-*terminal amino group of tripeptides (SRR-NH_2_, RSR-NH_2_, RRS-NH_2_) and the β-d-GlcNAc sugar was attached to Ser. The obtained glycol-lipopeptides displayed stronger activity against both planktonic and biofilm cultures of ESKAPE strains. However, their toxicity toward human erythrocytes was found to be correlated to the hydrophobicity and position of serine/glycosylated serine [96].

Moreover, among the various approaches, the combination of peptide cyclization and lipidation represents another strategy for enhancing the antimicrobial activity of AMPs. Jensen et al. introduced fatty acid moieties of different lengths and in diverse positions in a cyclic head-to-tail peptide called S3(B). They found that the introduction of fatty acids in positions adjacent to the flexible linker was more strongly correlated to an enhancement of antimicrobial activity, whereas these cyclo-lipopeptides became highly hemolytic when the carbon-chain length exceeded 10 (C10), overlapping with the optimum for antimicrobial activity (C8–C12). The authors found that the most promising candidate (C8) 5 showed similar antimicrobial activity to that of S3(B) and also featured an improved hemolytic profile [97].

Several lipopeptides with higher antimicrobial activity and moderate cytotoxicity have been isolated and identified from natural sources (Table 1). For example, daptomycin, produced and isolated from *Streptomyces roseosporus*, represents one of the most studied lipopeptides active against Gram-positive bacteria and multi-drug resistant pathogens [98]. Daptomycin is a thirteen-amino-acid peptide linked to a C10 fatty acid chain and was approved by the *Food and Drug Administration* (FDA) in 2003 for the treatment of skin and soft tissue infections caused by Gram-positive bacteria. The daptomycin mechanism consists of membrane permeabilization, the inhibition of cell wall synthesis, and the alternation of membrane fluidity and curvature. Unfortunately, its extensive clinical use has already led to resistance due to alterations in the membrane lipid composition such as a significant decrease in the amount of phosphatidylglycerol (PG) or an increase in cardiolipin (CL), which limits daptomycin’s action against bacteria [99].

Surfactin is a lipopeptide widely studied due to its antibacterial, antiviral, anti-adhesiveness, and anti-inflammatory properties. It is a cyclic lipopeptide (EL*l*VD*l*L) containing C16-β-hydroxy fatty acid linked through a lactone bond [100]. The main properties of surfactin are related to its interaction with the lipid components of membranes, which causes membrane depolarization [101].

Another lipopeptide is fengycin, a decapeptide bearing the C-16-β-hydroxy fatty acid in the *N-*terminus, which is produced by several *Bacillus subtilis* strains [102,103]. Despite being ineffective against yeast and bacteria, it has excellent antifungal properties and low hemolytic activity, making it suitable for dermatological applications.

Recently, five lipopeptides, namely peptidolipins B–F, were identified in a marine *Nocardia* sp. isolated from the ascidian *Trididemnum orbiculatum* [104]. Each peptidolipin has a lipid chain and, in particular, the isoforms B, C, and D have a lipid chain of C-23, C-25, and C-27, respectively, whereas the peptidolipins E and F contain the olefin and cyclopropyl moieties, respectively. Regardless of the chain length, peptidolipins B–F all exhibited moderate activity against methicillin-sensitive *S. aureus* (MSSA) and methicillin-resistant *S. aureus* (MRSA), with an MIC of 64 μg/mL.

Among the peptide-engineering strategies used to improve the antiviral activity of peptides, the addition of lipid moieties was demonstrated dramatically increase the antiviral potency. Cholesterol (Chol) tagging has been applied to an antiviral peptide derived from HIV-1 gp41. The resulting inhibitor is more potent (25- to 100-fold) than the clinical drug enfuvirtide (50- to 400-fold) [105]. Membrane targeting of antiviral peptides derived from the gB glycoprotein of *Herpes simplex virus* type 1 also depends on both the PEG linker length and cholesterol that determine the enhanced antiviral potency [106].

## 5. Applications of Glycosylation and Lipidation to Improve Activity through Enhancing Delivery

Recently, another challenging strategy used to develop antimicrobial therapeutics is the use of lipopeptides or carbohydrates to develop nanosystems that are decorated on their surface with AMPs [107].

Several lipopeptides were designed to develop supramolecular delivery systems for antimicrobial peptides. For example, Chen et al. developed antimicrobial supramolecular nanofibers with melittin as the AMP on their surface [108]. The delivery of melittin through this system decreased its cytotoxicity toward mammalian cells since in this nanosystem, the melittin was structurally constrained and was not entirely available for the interaction and permeability of the mammalian cell membrane. As a result, the melittin-based nanofibers exhibited high selectivity toward bacterial cells and demonstrated good antimicrobial potency.

Another example of AMP-based nanofibers was presented by Lombardi and co-workers [109]. The authors engineered nanofibers using lipopeptide PAs containing an aliphatic polyalanine sequence and a lipidic tail of C-19 [109,110]. The nanofibers’ surface decorated with the peptide WMR (derived from Myxinidin) as an AMP exhibited a significant ability to deliver WMR, induce a strong inhibition of biofilm formation, and eradicate the already formed biofilms of *P. aeruginosa* and *C. albicans*.

However, another strategy used to improve the efficacy of AMPs involves using carbohydrates as building blocks to develop AMP-based delivery systems. An approach widely used is the conjugation of chitosan to AMPs or the preparation of chitosan-based nanosystems loaded with AMPs. Chitosan is a natural and biocompatible polymer composed of β-1,4-linked d-glucosamine and N-acetyl-d-glucosamine, which is obtained through the deacetylation of the polymer chitin. An example is anoplin–chitosan conjugates, which were synthesized by Sahariah et al. through the CuAAC reaction to improve the therapeutic index of anoplin, an AMP isolated from the venom of the solitary wasp [111]. The grafting of anoplin with chitosan improved its activity against Gram-positive and Gram-negative bacteria, thereby removing its strong hemolytic effect on red blood cells.

In addition, carbohydrates can be used to develop nanosystems with AMPs loaded into their core. Chitosan-based nanoparticles (CS-NPs) are widely used for AMP delivery as they have an encapsulation efficacy of up to 98%, have high cytocompatibility, and can achieve the sustained release of AMPs. For instance, the encapsulation of temporin B, a natural AMP isolated from amphibian skin secretions, into CS-NPs, resulted in enhanced and sustained antimicrobial activity against several strains of S. epidermidis for 4 days, demonstrating a long-lasting antibacterial effect [112]. A similar study was performed on the synthetic peptide Octominin derived from the defense protein of Octopus minor (Fish Shellfish Immunol. 2021, 110, [23,24,25,26,27,28,29,30,31,32,33,34]). As in the case of temporin B, the encapsulation of Octominin into the core-shell of CS-NPs resulted in the sustained release of Octominin over 96 h, causing higher biofilm inhibition and the eradication of *C. albicans* and Acinetobacter baumanii [113]. In addition, CS-NPs loaded with Octominin showed reduced toxicity in comparison to free Octominin both in vitro using human embryonic kidney 293 (HEK 293) cells and in an in vivo Zebrafish model.

Interestingly, glycans play a crucial role in targeting infections of the CNS [114]. For instance, O-glycosylation by conjugating sialic acid allows for the production of BBB-penetrating molecules that are able to bypass the BBB and eliminate persistent infections. In addition, O-linked glycosylation has been shown to be effective in improving BBB penetration of opioid peptides, thereby increasing their serum and brain stability [115].

## 6. Conclusions Remarks

The rapid increase in antibiotic resistance has generated huge interest in AMPs as potential alternatives to combat multidrug-resistant bacteria due to their broad effectiveness at low concentrations against many species. Unfortunately, their use is clinically limited by their reduced bioavailability and serum stability in vivo.

Here, we emphasized that glycosylation and lipidation can be considered potent strategies for improving AMP efficacy and overcoming many of their limitations.

One issue with AMPs is their broad-spectrum activity, which can lead to a reduction in selectivity between mammalian and bacterial membranes and an increase in cytotoxicity. Another drawback is their immunogenicity, which can be addressed by decorating them with glycans derived from bacteria such as polysialic acid to decrease their immunoreactivity within the host. In this context, the glycosylation strategy could mask the immunogenicity of AMPs, for instance, the conjugation of sialic acid residues could reduce the recognition of AMPs by T cell and B cell receptors.

Moreover, as described, the glycosylation of both natural and synthetic AMPs influences their antimicrobial activity, cytotoxicity, and target specificity. The various glycosylation strategies, including *O-*, *N-*, *C-*, and *S-*glycosylation, involve conjugating a sugar moiety such as glucose to an AMP, with GalNAc bearing the specific functional group depending on the chosen strategy. Glycosylation can induce an increase in the rigidity and proteolytic stability of AMPs, as well as cause secondary structure changes. *N-* and *O-*glycosylation are similar to PEGylation as they protect AMPs from rapid renal clearance, but glycosylation is preferable because it is safer compared to the introduction of a synthetic polymer. In addition, the conjugation of a carbohydrate to AMPs or its encapsulation in carbohydrate-based nanosystems is a strategy that is used to ensure the delivery of AMPs and improve their pharmacokinetic and pharmacodynamic properties. One of the most widely used vectors is chitosan-based nanoparticles, which exhibit a high encapsulation efficacy and a strong capacity to both improve antibacterial activity through sustained release and reduce selectivity against mammalian cells.

Likewise, lipidation influences the physicochemical properties of AMPs, including their hydrophobicity and self-assembling ability, because the incorporation of long lipid tails can induce peptide aggregation in solution, thereby drastically influencing the antimicrobial activity of AMPs. Indeed, it is crucial to maintain the right balance between hydrophilic and hydrophobic domains in peptide sequences to ensure interactions with the bacterial membrane while maintaining poor selectivity toward eukaryotic cells. Moreover, the long lipid tails linked to the *N-* or *C-* terminus of AMPs increase their affinity with bacterial membranes, facilitating their insertion into lipid bilayers and subsequent membrane curvature and disruption. Additionally, lipopeptides are used as building blocks to develop supramolecular nanosystems such as nanofibers or nanotubes for delivering AMPs. Generally, these nanostructures are engineered specifically for the AMP because they are modifiable and can be customized for specific bacterial infections.

Overall, the de novo design of lipidated and glycosylated peptides represents a valuable strategy for developing meaningful alternatives to antibiotics, offering many advantages over current strategies. The significant benefits provided by glycosylation and lipidation of therapeutic peptides highlight the potential to enhance the therapeutic index of traditional AMPs.

## Figures and Tables

**Table 1:** Examples of natural lipopeptides and their activity.

Lipopeptide	Source	Chain Length	Activity
Daptomycin	*Streptomyces roseosporus*	C10	Antibacterial
Surfactin	*Bacillus subtilis*	C16	Antibacterial, antiviral
Fengycin	*Bacillus subtilis*	C16,	Fungicide
Peptidolipins B–F	*Nocardia* sp.	C23, C25, C27, olefin cyclopropane	Antibacterial
Friulimicin B	*Actinoplanes friuliensis*	C14	Antibacterial
Arylomycin A2	*Streptomyces* sp.	C12	Antibacterial
Globomycin	*Streptomyces hagronensis*	C6	Antibacterial
Tsushimycin	*Streptomyces* sp.	C14	Antitrypanosomal

## Data Availability

Not applicable.
